# Exploring the Potentiality of Native Actinobacteria to Combat the Chilli Fruit Rot Pathogens under Post-Harvest Pathosystem

**DOI:** 10.3390/life13020426

**Published:** 2023-02-02

**Authors:** Rajamuthu Renuka, Kupusamy Prabakar, Rangasamy Anandham, Lakshmanan Pugalendhi, Lingam Rajendran, Thiruvengadam Raguchander, Gandhi Karthikeyan

**Affiliations:** 1 Department of Plant Pathology, Pandit Jawaharlal Nehru College of Agriculture and Research Institute, Karaikal 609603, India; 2 Department of Plant Pathology, Centre for Plant Protection Studies, Tamil Nadu Agricultural University, Coimbatore 641003, India; 3 Department of Agricultural Microbiology, Directorate of Natural Resource Management, Tamil Nadu Agricultural University, Coimbatore 641003, India; 4 Department of Vegetable Science, Horticultural College and Research Institute, Tamil Nadu Agricultural University, Coimbatore 641003, India

**Keywords:** actinobacteria, *Streptomyces tuirus*, chilli fruit rot, *Colletotrichum scovillei*, *Colletotrichum truncatum*, *Fusarium oxysporum*, liquid bio-formulation

## Abstract

Chilli is an universal spice cum solanaceous vegetable crop rich in vitamin A, vitamin C, capsaicin and capsanthin. Its cultivation is highly threatened by fruit rot disease which cause yield loss as high as 80–100% under congenial environment conditions. Currently actinobacteria are considered as eco-friendly alternatives to synthetic fungicides at pre and post-harvest pathosystems. Hence, this research work focuses on the exploitation of rhizospheric, phyllospheric and endophytic actinobacteria associated with chilli plants for their antagonistic activity against fruit rot pathogens *viz*., *Colletotrichum scovillei*, *Colletotrichum truncatum* and *Fusarium oxysporum*. In vitro bioassays revealed that the actinobacterial isolate AR26 was found to be the most potent antagonist with multifarious biocontrol mechanisms such as production of volatile, non-volatile, thermostable compounds, siderophores, extracellular lytic enzymes. 16S rRNA gene sequence confirmed that the isolate AR26 belongs to *Streptomyces tuirus*. The results of detached fruit assay revealed that application of liquid bio-formulation of *Stretomyces tuirus* @ 10 mL/L concentration completely inhibited the development of fruit rot symptoms in pepper fruits compared to methanol extracts. Hence, the present research work have a great scope for evaluating the biocontrol potential of native *S. tuirus* AR26 against chilli fruit rot disease under field condition as well against a broad spectrum of post-harvest plant pathogens.

## 1. Introduction

Chilli (*Capsicum annuum* L.) is one of the most economically important spices cum solanaceous vegetable crops and is grown throughout the world for its green and red ripe fruits. It is a universal spice crop of India and occupies a major share in the Indian economy. In addition to adding pungency, taste, aroma and colour to cuisines, chilli have been used for centuries as medicine with countless health benefits, with antioxidant, anti-mutagenic, anti-carcinogenic, anti-arthritic and anti-inflammatory properties. Chilli fruits are rich in capsaicin, an appetite stimulant, and capsanthin, a pigment that gives its distinctive flavour and colour. Green chilli fruits contain more vitamin C than citrus fruits, whereas red chilli fruits contain more vitamin A than carrots [1,2]. Despite its rich nutritional and economic value, its commercial production is greatly threatened by fruit rot disease caused by complex pathogens including different species of *Colletotrichum*, *Fusarium* and *Alternaria* [3,4,5]. These pathogens extensively damage the fruits and significantly reduce the quality, yield, appearance and marketability of the fruits [6,7]. It is a highly destructive pre- and post-harvest disease which causes yield losses up to 100% under congenial environmental conditions [8,9,10].

Though this disease can be managed with the repeated application of fungicides, pre- and post-harvest application of synthetic fungicides has been curtailed due to the persistence of fungicides on the fruits, which pose a direct risk to consumers and the environment through food chain contamination [11,12,13]. The use of naturally occurring bioactive compounds, especially those derived from antagonistic microorganisms, have been explored as prospective alternatives to synthetic fungicides due to their reduced toxicity and impact on humans and the environment [14,15,16]. Biological control of chilli anthracnose using antagonistic microorganisms or their metabolites is not a new concept, but a sustainable and ecologically acceptable approach in the context of leaving no toxic residues on the produce, safer application methods and ease of delivery, with minimal reliance on chemicals [17,18]. 

Although different groups of microorganisms have been employed for disease management, several research findings over the past few decades have highlighted the biocontrol potential of actinobacteria against a wide range of plant pathogens [19,20,21] through various mechanisms, including fungal cell-wall lysis, antibiosis, competition for nutrients, induction of host systemic resistance, phytotoxin degradation, plant growth stimulation, nutrient assimilation, rhizosphere competence and mineral availability [22,23,24,25,26,27,28].

Several species of actinobacteria were reported to have strong antagonistic activity against various species of *Colletotrichum* infecting a variety of crops. Taechowisan et al. [29] reported that *Streptomyces* spp. SRM1 exhibited antagonistic activity against *Colletotrichum musae* causing anthracnose in banana. *Streptomyces violaceoruber* reduced the incidence of chilli anthracnose by inhibiting the spore germination and mycelial growth of *Colletotrichum capsici* [30]. *Streptomyces ambofacines* S2 extract completely inhibited the expression of anthracnose symptoms of *Colletotrichum gloeosporioides* in red pepper fruits [18]. Actinobacteria not only prevent post-harvest pathogenic infection but also prolong the shelf life of a variety of crops without upsetting the natural balance.

A diverse group of actinobacteria inhabit the rhizosphere, phyllosphere and endosphere region of the plant and a subset of these provide a wide range of services and benefits to the plant in terms of suppressing plant diseases, promoting plant growth, increasing crop yield and enhancing soil fertility [31,32,33]. Particularly, when employed to curtail the plant infections, native actinobacterial isolates are more adaptable to their regular niche, have a higher success rate and are more resilient to local environmental challenges than the introduced microbes [34,35]. Furthermore, the introduced microbes must be able to co-habit with the native microbiome in order to provide more benefits to the plants. Hence, precolonization of the host by well adapted native biocontrol agents may prevent the growth and survival of plant pathogens.

The present study was, therefore, undertaken with the following objectives: (1) to isolate native actinobacterial isolates associated with rhizosphere, phyllosphere and surface sterilized tissues of chilli plants; (2) to identify the efficient actinobacterial isolate having antifungal potential against fruit rot pathogens *Colletotrichum* spp., and *Fusarium* sp.; (3) to unravel the antifungal mechanisms of potential actinobacterial isolates against fruit rot pathogens under in vitro conditions; (4) to assess the in vivo antifungal efficacy of liquid formulation and soluble metabolites of potential actinobacterial isolates on chilli fruits.

## 2. Materials and Methods

### 2.1. Fruit Rot Pathogens

Fruit rot fungal pathogens *viz*., *Colletotrichum scovillei*, *Colletotrichum truncatum* and *Fusarium oxysporum* were isolated from infected chilli fruits collected from various locations of Tamil Nadu, India. The infected portion of the fruits were cut into small pieces (5 mm) using a sterile blade and surface sterilized with 1% NaOCl_4_ for 1–2 min followed by 70% ethanol for 30 s and rinsed thrice with sterile distilled water [36]. The surface disinfected fruit pieces were placed onto sterile Potato Dextrose Agar (PDA) medium amended with streptomycin sulphate (0.03 g L^−1^) and incubated at 28 ± 2 °C for 7 days. Pure cultures of the pathogens were obtained by single hyphal tip method. The stock cultures of the pathogens were maintained as pure cultures on PDA slants at 4 °C.

### 2.2. Antagonistic Actinobacteria

Rhizosphere actinobacteria were isolated from the rhizosphere soil of healthy chilli plants as described by Anwar et al. [37]. The soil samples were taken from a depth of 10–20 cm and subjected to dry heat pre-treatment for 4 h at 45 °C [38] to diminish the fast growing and abundant soil bacteria that would hinder slow growing actinobacteria [39]. Ten grams (10 g) of pre-treated soil was suspended in 90 mL of sterile distilled water, shaken thoroughly for 1 h at 100 rpm in an orbital shaker and allowed to settle for an hour. Subsequently, samples were serially diluted up to 10^−5^ dilutions and 1 mL aliquot from 10^−3^–10^−5^ dilutions were plated on sterile Starch Casein Agar (SCA) supplemented with 25 µg/mL nalidixic acid and 50 µg/mL nystatin as antibacterial and antifungal agents [40].

Phyllospheric actinobacteria were isolated from the leaf, stem, flower and fruits of healthy chilli plants [41]. Ten grams (10 g) of samples were preheated at 70 °C for 15 min and transferred to 90 mL of 0.85% saline buffer (NaCl) and kept in an orbital shaker at 250 rpm for 30 min at 28 ± 2 °C. The solution thus obtained was subjected to the standard serial dilution pour plate technique on Starch Casein Agar (SCA) supplemented with nalidixic acid (25 µg/mL) and nystatin (50 µg/mL).

The actinobacteria from the surface sterilized plant tissues were isolated as per the procedure described by Li et al. [42]. A five-step procedure was employed for the sterilization of the plant tissues: (i) the tissue segments were surface-sterilized in 0.1% sterile Tween 20 for 1 min, (ii) the samples were sterilized with 5% sodium hypochlorite for 4 min (leaf samples) or 6 min (stem and root samples), (iii) the samples were then rinsed in 2.5% (*w*/*v*) sodium thiosulfate for 10 min and washed three times with sterile distilled H_2_O, followed by (iv) immersing in 70% (*v*/*v*) ethanol for 4 min (leaf samples) or 6 min (stem and root samples), and finally (v) the samples were washed with sterile distilled water for a minimum of three times. To validate the successful surface disinfection process, 0.2 mL of water from the final wash was spread onto the isolation medium and incubated at 28 ± 2 °C. One gram of surface-sterilized plant tissues was homogenized in a mortar and pestle with 1 mL of 0.9% saline buffer (*w*/*v*). One millilitre of the tissue suspension was serially diluted and 10^−3^–10^−5^ dilutions were plated on Starch Casein Agar plates. The plates were incubated at 28 ± 2 °C for 7–10 days. Powdery, bright actinobacterial colonies were purified, suspended in 20% glycerol and stored at −80 °C as stock culture [43].

### 2.3. In Vitro Antifungal Bioassay

#### 2.3.1. Primary Screening of Actinobacterial Isolates for the Antifungal Activity against Chilli Fruit Rot Pathogens

Fifty-two actinobacterial isolates were screened for their antifungal activity against chilli fruit rot pathogens by dual-culture assay [44]. The test isolates were streaked at one corner of the PDA plates (10 mm from the periphery of a 90 mm diameter Petri dish) and incubated at 28 ± 2 °C for 4 days. After incubation, the 5-day-old pathogen fungal disc was placed opposite to actinobacterial streak (10 mm away from the periphery). Petri dishes without actinobacterial isolates served as the control. All plates were incubated at 28 ± 2 °C for 7 days. All the isolates were tested in triplicate. After incubation, the zone of inhibition was measured and the per cent inhibition of mycelial growth was calculated. The zone of inhibition (ZI) was measured as the diameter of the halo zone (in cm) between the actinobacteria and pathogen colony as and when the pathogen in the control plate covered the entire plate. Per cent inhibition of mycelial growth (PIMG) was determined according to the formula: PIMG = (C − T)/C 100, where C and T are the mycelial growth of pathogenic fungus in the control plate and dual culture plate, respectively. The degree of antifungal activity of various actinobacterial isolates against the tested pathogens were evaluated based on the zone of inhibition (ZI) (in cm) and per cent inhibition of mycelial growth (PIMG) [45]. Based on the zone of inhibition, the antagonistic activity of actinobacterial isolates were grouped into four categories according to Lee and Hwang [46] as: − no inhibition (ZI ≤ 0); + weak inhibition (ZI = 0.1–1.0 cm); ++ moderate inhibition (ZI = 1.01–2 cm); and +++ strong inhibition (ZI ≥ 2 cm).

#### 2.3.2. Secondary Screening for the Antifungal Activity of Actinobacterial Isolates

The antifungal activity of six actinobacterial isolates which exhibited the strongest inhibition against the tested pathogens by dual culture assay was further confirmed by paired culture antibiosis assay as per the protocol of Liotti et al. [47] with slight modification. An 8 mm mycelial disc of the pathogen was placed at the centre of a Petri dish containing PDA medium and the actinobacterial isolate was streaked at equidistance on both sides of the pathogen, about 10 mm from the periphery of Petri dish. A control plate was maintained without actinobacteria. The experiment was replicated thrice. After 7 days of incubation at 28 ± 2 °C, the percentage inhibition of mycelial growth (PIMG) of the pathogen was calculated as per the formula described above.

#### 2.3.3. In Vitro Screening of Actinobacterial Isolates for Production of Extracellular Lytic Enzymes and Siderophore

The actinobacterial isolates were assayed for their biocontrol traits *viz*., amylase, cellulase, chitinase and protease production by spot inoculating 10 μL of culture in starch agar medium [48], Carboxy Methyl Cellulose (CMC) agar medium [49], colloidal chitin agar medium [50] and skim milk agar medium [51], containing starch, cellulose, colloidal chitin and casein as the respective substrates. Siderophore production was assayed on Chrome Azurol Sulphonate (CAS) agar medium according to the methodology of Sadeghi et al. [52]. The plates were incubated for 5–7 days at 28 ± 2 °C. Three replications were maintained for each actinobacterial isolate.

The amylase activity of the actinobacteria was evaluated by flooding the plate with Lugol’s iodine solution for 30 s. A clear hydrolysis zone around the colonies against the blue background indicated the hydrolysis of starch by the amylase enzyme [53]. Cellulase activity was determined by flooding the plates with 0.1% Congo red solution and counter staining with 1 M NaCl for 15–20 min. The formation of a clear zone around the colony due to the hydrolysis of cellulose indicated a positive result for the production of the cellulase enzyme by the actinobacterial isolates. The isolates positive for chitinolytic and proteolytic activity produced a clear halo zone around the colonies due to the hydrolysis of chitin and casein in the respective media. The formation of a yellow to orange halo around the actinobacterial colonies due to the removal of iron from CAS represented a positive result for the production of siderophore.

#### 2.3.4. Antifungal Activity of Volatile Organic Compounds

The four most active actinobacterial isolates that showed significant positive results for the production of cell wall-degrading enzymes and siderophore were subjected to additional assays on the production of volatile, non-volatile and thermostable compounds. The antifungal activity of volatile organic compounds (VOCs) produced by the actinobacterial isolates was tested against the fruit rot pathogens by the double-sealed plate method [54]. A 90 mm diameter Petri dish bottom containing 15 mL of ISP4 medium was streaked with a loopful of actinobacterial culture. An 8 mm diameter mycelial plug of the pathogen was inoculated in the centre of another Petri dish bottom containing 15 mL of potato dextrose agar medium. A Petri dish “sandwich” was made with the antagonist Petri dish placed over the pathogen plate in such a way that the pathogen plate was at the bottom and antagonist plate was on the top. The sandwiched Petri dish was sealed together with a parafilm without any gaps and incubated at 28 ± 2 °C for 7–10 days. A Petri dish containing ISP4 medium without the antagonist placed over the pathogen plate served as the control. The parafilm-sealed plates ensured no physical contact between the pathogen and antagonist. The experiment was conducted with three replications for each isolate. The rate of inhibition (%) of mycelial growth was calculated as described previously.

#### 2.3.5. Antifungal Activity of Non-Volatile Metabolites

The antifungal activity of non-volatile metabolites in the cell-free culture filtrate of actinobacterial isolates was determined using the seeded agar method [55,56]. Actinobacterial isolates were cultured in a 250 mL conical flask containing 100 mL ISP4 broth and incubated in an orbital shaker at 150 rpm for 7 days at 28 ± 2 °C. Then the culture broth was centrifuged at 10,000 rpm for 15 min at 4 °C. The supernatant obtained was filtered through a 0.22 μm nitrocellulose membrane filter to obtain cell-free culture filtrate and subjected to an antifungal assay. The filtrate was mixed with warm PDA (25%) and plated in a sterile Petri dish. Finally, an 8 mm mycelial disc of the pathogen was placed at the centre of the seeded PDA medium in the Petri dish. The pathogen growth on the Petri dish without the cell-free culture filtrate served as the control. The plates were incubated at 28 ± 2 °C until the mycelial disc in the control plate completely covers the plate. Three replicates were maintained for each isolate. Per cent inhibition (PI) of mycelial growth was calculated as described previously.

#### 2.3.6. Antifungal Activity of Thermostable Compounds

The actinobacterial isolates were cultured in 100 mL ISP4 broth in a 250 mL conical flask with constant agitation in an orbital shaker (150 rpm) for 7 days at 28 ± 2 °C. The actinobacterial cells were harvested by centrifugation at 10,000 rpm for 15 min. Twenty-five millilitres of the supernatant were transferred to a conical flask containing 75 mL PDA medium and sterilized at 121 °C for 20 min. The actinobacterial metabolite-amended sterile medium was plated into Petri dish and a 9 mm mycelial disc of the tested pathogen was placed at the centre of solidified medium. The pathogen growth on PDA medium without actinobacterial metabolite served as the control. The Petri dishes were incubated at room temperature for 7 days and the Per cent Inhibition (PI) of mycelial growth of the pathogen was assessed as per the formula described above.

#### 2.3.7. Assessment of In Vitro Antifungal Traits

Among the six isolates, the best isolate with the highest antagonistic potential was selected based on a bonitur scale as described by Passari et al. [57] and El-Sayed et al. [58]. In this scale, points were given for each in vitro antifungal trait and the maximum bonitur score is 24 points. The per cent inhibition of mycelial growth (PIMG) was evaluated as follows: if PIMG is 30–54.9% = 1 point; 55–74.9% = 2 points; 75–95% = 3 points. Lytic enzyme production was evaluated with 1 point and siderophore with 2 points each.

### 2.4. Scanning Electron Microscopy (SEM)

The interaction of the actinobacterial isolate AR26 which exhibited strong antifungal activity against the pathogens in the dual culture plate was documented by Scanning Electron Microscope (SEM) (Model: FAI QUANTA 250, Czech Republic) at 15 KV [59]. Mycelial discs (5 mm) of the pathogen from the periphery of inhibition zone in the dual culture plate as well as in the control plate were cut with a sterile scalpel and transferred to perforated capsules and fixed in 1.5% glutaraldehyde in phosphate buffer for 4 h [60]. Then, the specimens were washed with 0.2 M sodium cacodylate buffer (pH 6.2) and dehydrated with an increasing concentration of ethanol washes from 0–100% at 10 min intervals (0%, 30%, 50%, 70%, 80%, 90% and 100%). Later the specimens were mounted on aluminium stubs using conductive double-sided carbon tape. The stubs were then lyophilized, and sputter coated with gold (5 nm thickness). Finally, any morphological changes of the pathogen mycelium in the dual culture plate as well as in the control plate were examined under scanning electron microscope.

### 2.5. Molecular Characterization of Actinobacterial Isolates

The genomic DNA of the actinobacterial isolate was extracted from the spore masses using the Cetyl Trimethyl Ammonium Bromide (CTAB) method [61]. The 1.5 kb full length 16S rRNA gene of actinobacteria was amplified by Polymerase Chain reaction (PCR) with a forward primer 27F (5′ AGAGTTTGATCCTGGCTCAG-3′) and reverse primer 1492R (5′-GGTTACCTTGTTACGACTT-3′) [62]. The PCR amplification was performed with a 25 μL reaction mixture which contained 10 μL of master mix, 1 μL of bacterial genomic DNA at a concentration of 20 ng, 1 μL of each primer at a concentration of 10 pM and 12 μL of sterilized deionized water. The PCR amplification conditions included an initial denaturation at 94 °C for 5 min, 35 cycles of denaturation at 94 °C for 1 min, annealing at 55 °C for 1 min, extension at 72 °C for 40 s and final extension at 72 °C for 10 min. The PCR amplified products were visualized on 1% agarose gel with a UV transilluminator and photographed using the gel documentation system and sequenced at Biokart India Pvt. Ltd., Bangalore, India. The sequence similarities were determined by BLAST analysis (Basic Local Alignment Search Tool) (BLAST, (https://www.ncbi.nlm.nih.gov) (accessed on 27 November 2022) and submitted in GenBank. The most homologous sequences showing the highest similarity were retrieved from the NCBI GenBank database and multiple sequences were aligned using the ClustalW algorithm. A phylogenetic tree was constructed with closely related nucleotide sequences using the Neighbour-Joining (NJ) method [63] using MEGA (Molecular Evolutionary Genetics Analysis) 11 software [64] with bootstrap values of 1000. Evolutionary distances were calculated using the maximum combined likelihood method and are given in units of the number of base substitutions per site.

### 2.6. Antifungal Bioassay of Liquid Formulation of Actinobacterial Isolate on Chilli Fruits

The antifungal activity of actinobacterial isolate AR26 on green chilli fruits was determined as per the antifungal bioassay of Liottia et al. [47]. Fresh fruits of the chilli hybrid “Ganga” of uniform size and maturity without wounds, scars and rots on their surface were surface sterilized as described previously. The surface sterilized green chilli fruits were wounded to the depth of 1 mm with a sterile needle and subjected to following treatments. (i) healthy control: chilli fruits were inoculated with 20 μL of sterile distilled water, (ii) pathogen-inoculated control: chilli fruits were inoculated with 6 mm mycelial disc of pathogen culture, (iii) antagonist inoculated control: chilli fruits were inoculated with 20 μL of liquid formulation of *S. tuirus* AR26 at10 mL/L containing 9 × 10^8^ CFU/mL, (iv) chilli fruits were first inoculated with 20 μL of liquid formulation of *S. tuirus* at 5 mL/L and after an hour of incubation, 6 mm mycelial disc of respective pathogens were placed over it, (v) chilli fruits were first inoculated with 20 μL of liquid formulation of *S. tuirus* at 10 mL/L and 6 mm mycelial disc of respective pathogens were placed over it.

Inoculated fruits of each treatment were placed in separate glass Petri dishes, sealed with parafilm and incubated at 28 ± 2 °C for 7 days. The experiment was conducted statistically as a completely randomized design (CRD) in three replicates of five fruits each. The progress of the symptom on the fruits was measured as the lesion diameter after seven days of incubation. The percentage of inhibition of fruit rot symptom and disease incidence was calculated as per the formula given below. Per cent disease reduction = [(D − d) × 100]/D, where D is the lesion diameter in pathogen-inoculated control fruits, and d is the lesion diameter in actinobacteria and pathogen co-inoculated fruits.

Disease incidence = (Number of diseased chili fruits/Total number of chili fruits) × 100.

### 2.7. Antifungal Bioassay of Soluble Metabolites of Actinobacterial Isolate on Chilli Fruits

This assay was conducted to differentiate whether the antifungal activity was mediated by the presence of actinobacterial culture or by its metabolites. The soluble metabolites produced by the isolate AR26 in the dual culture plate in PDA medium were extracted from the zone of inhibition by excising the PDA medium from the inhibition zone using a sterile scalpel. Excised PDA medium was blended with HPLC-grade acetonitrile in a 1:4 ratio (5 g agar in 20 mL of HPLC grade acetonitrile). The mixture was incubated overnight at 28 ± 2 °C in an orbital shaker at 150 rpm. The homogenised samples were subjected to 10 min centrifugation at 10,000 rpm, and then filtered through Whatman No.1 filter paper to separate the agar particles and supernatant. The supernatant was dried in a vacuum flash evaporator (Roteva Equitron Make). After discarding the eluent, the final product was diluted in 1 mL of HPLC-grade methanol [65]. The extract obtained was tested for its ability to control chilli fruit rot pathogens. The assay was performed as described above with four treatments, using 20 μL of methanol extract of *S. tuirus* AR26 for treatments and 20 μL of methanol alone for control.

### 2.8. Statistical Analysis

The data was subjected to a single factor test of significance (ANOVA) using the analytical software SPSS version 16.0. Significant differences between the average values of each treatment (*p* ≤ 0.05) were determined using critical difference.

## 3. Results

### 3.1. Primary Screening for Antifungal Activity of Actinobacterial Isolates

**Figure 1:**
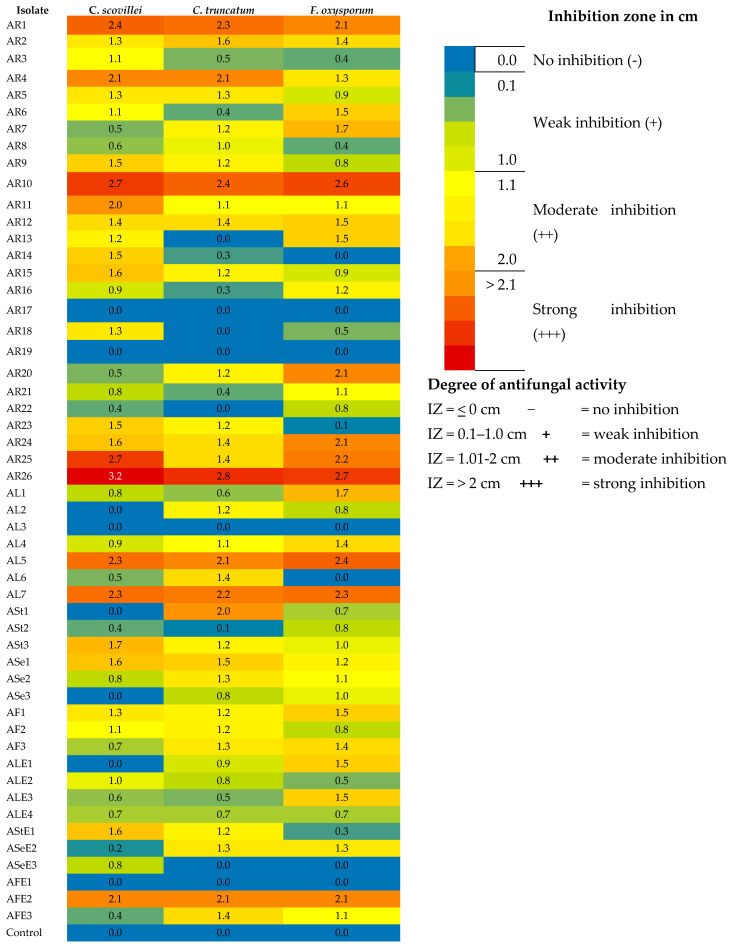
Heat map showing the in vitro antagonism of actinobacterial isolates against chilli fruit rot pathogens as represented by zone of inhibition.

**Figure 2:**
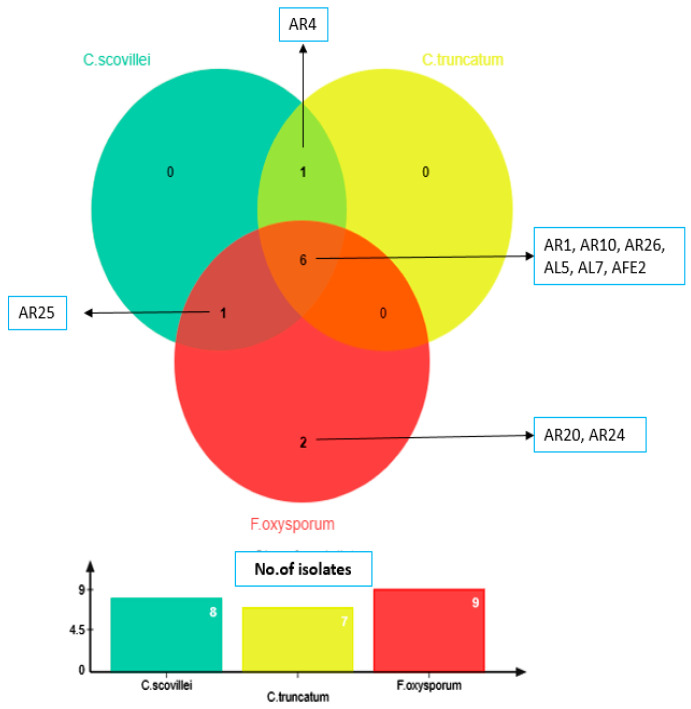
Venn diagram representing the actinobacterial isolates exhibiting strong antagonism against fruit rot pathogens.

**Figure 3:**
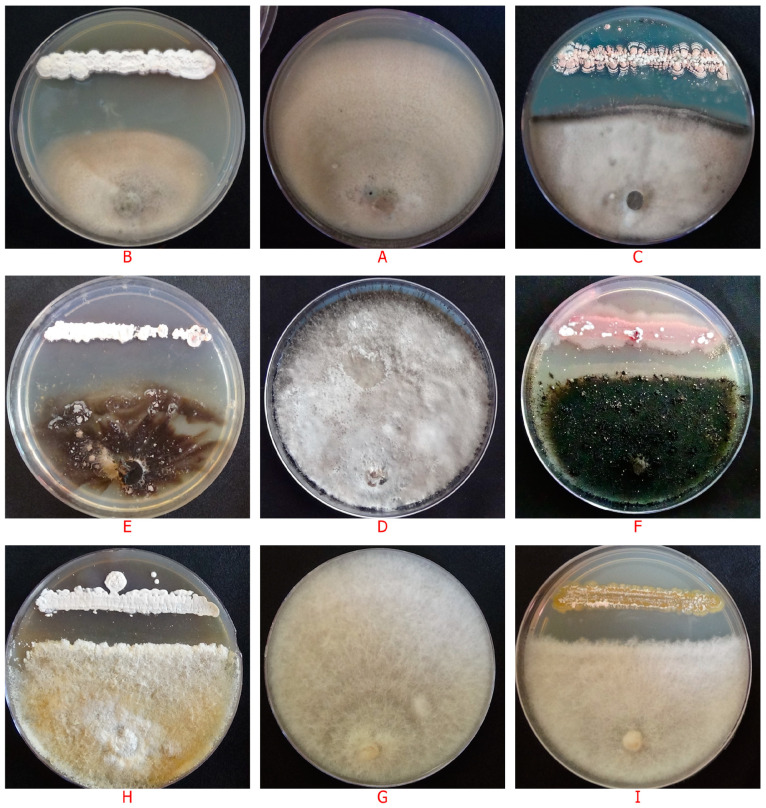
Antifungal activity of actinobacterial isolates against chilli fruit rot pathogens. (**A**) Control *C. scovillei* (**D**) Control *C. truncatum* (**G**) Control *F. oxysporum.* Antifungal activity of actinobacterial isolate AR26 against (**B**) *C. scovillei* (**E**) *C. truncatum* (**H**) *F. oxysporum*. Antifungal activity of actinobacterial isolate AR10 against (**C**) *C. scovillei* (**F**) *C. truncatum* (**I**) *F. oxysporum*.

In this study, 52 actinobacterial isolates obtained from rhizospheric (26), phyllospheric (16) and surface sterilized plant tissues (10) of chilli plants were screened for their antagonistic potential against chilli fruit rot pathogens *viz*., *C. scovillei*, *C. truncatum* and *F. oxysporum* by dual culture technique. About 19.2% of the rhizospheric isolates, 12.5% of phyllospheric isolates and 10.0% of the endophytic isolates exhibited strong antifungal activity against *C. scovillei*, whereas 15.4% of the rhizospheric isolates and 12.5% of the phyllospheric isolates and again 10.0 % of endophytic isolates showed antifungal activity against *C. truncatum*. With regard to *F. oxysporum*, 23.1% of rhizospheric, 12.5% of phyllospheric isolates and 10.0% of endophytic isolates showed strong antagonistic activity. Thirty-eight (73.07%) out of 52 isolates inhibited the mycelial growth of at least one out of the three pathogens with varying degrees of inhibitory action, ranging from 4.82% to 67.90% (weak to strong inhibition) (Supplementary Table S1 and Figure 1). Six isolates designated as AR1, AR10, AR26, AL5, AL7, and AFE2 strongly inhibited the growth of all three pathogens (Figure 2) with an inhibition zone (ZI) greater than 2 cm. Isolate AR26 was found to be significantly superior to other isolates, with the highest mycelial growth inhibition of 67.90%, 63.21%, and 60.37% and inhibition zones of 3.2 cm, 2.8 cm, and 2.7 cm respectively for *C. scovillei*, *C. truncatum*, and *F. oxysporum*, followed by the isolate AR10 (Figure 3).

### 3.2. Secondary Screening for the Antifungal Activity of Actinobacterial Isolates and Scanning Electron Microscopic Assay

**Figure 4:**
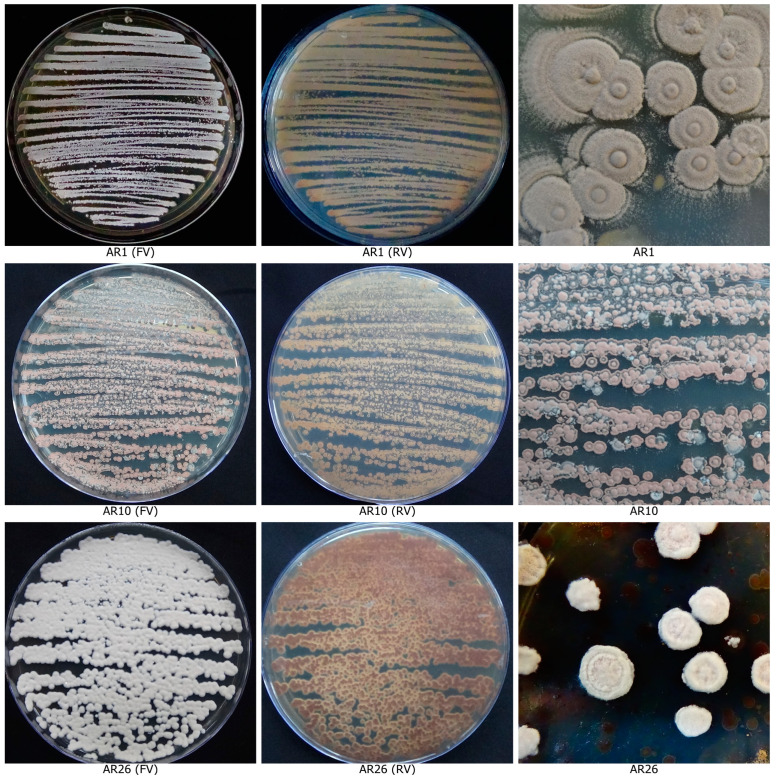

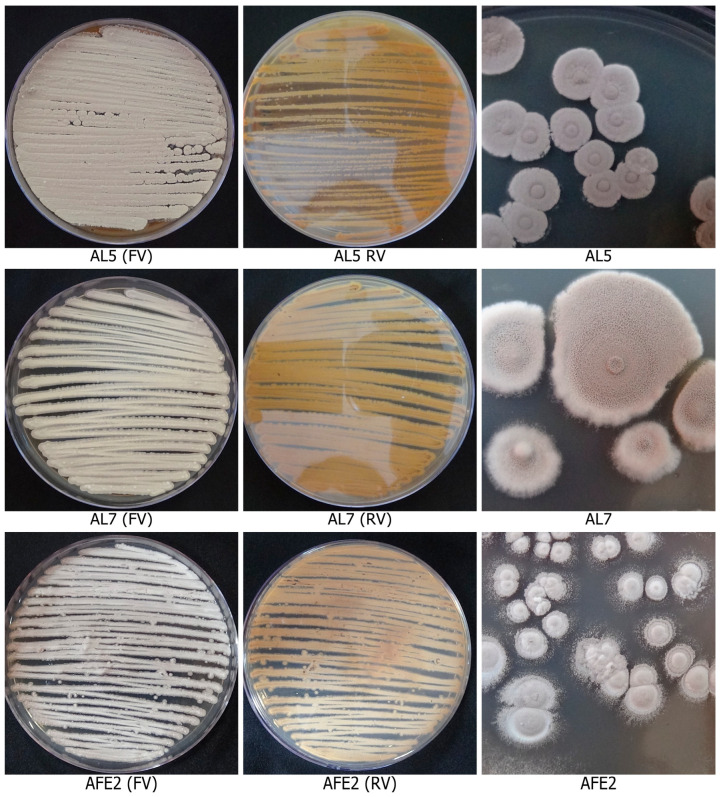
Actinobacterial isolates with potent antagonistic activity against fruit rot pathogens.

**Figure 5:**
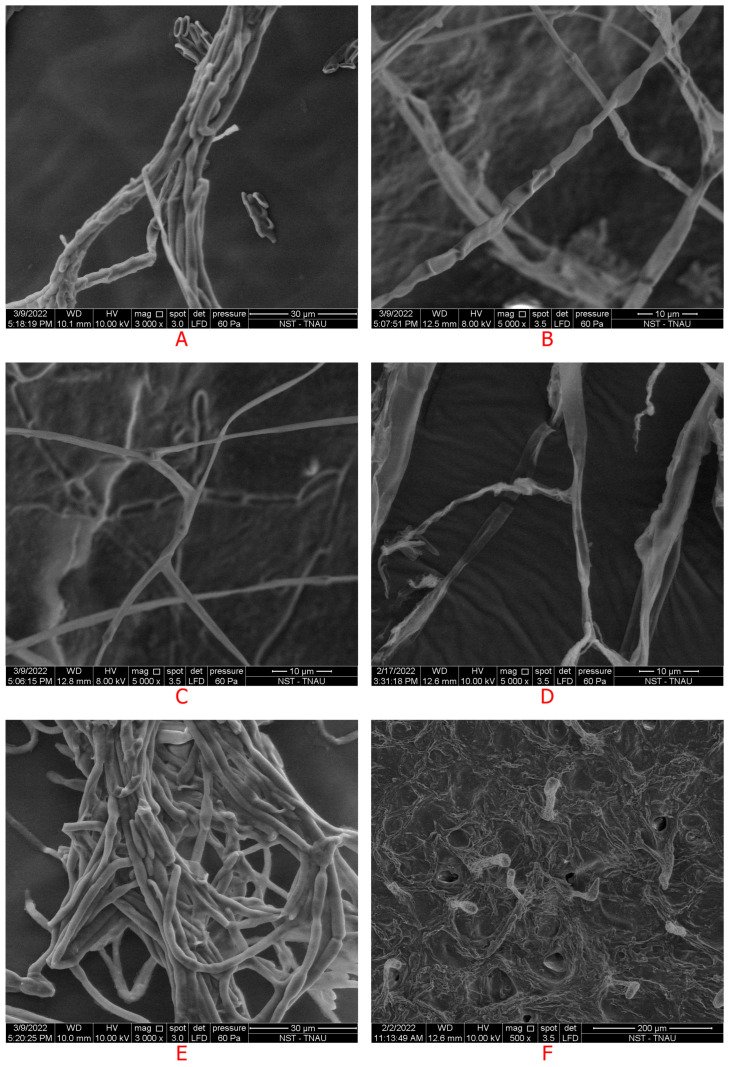
Scanning Electron Micrographs showing the interaction of antagonist. *S. tuirus* AR26 with *C. scovillei*, *C. truncatum* and *F. oxysporum*; *(***A**) intact mycelium of *C. scovillei* in the absence of antagonist; (**B**) twisted and shrunken hyphae of *C. scovillei* in the presence of antagonist; (**C**) mycelium of *C. truncatum* in absence of antagonist; (**D**) distorted hyphae of *C. truncatum* in the presence of antagonist; (**E**) dense and intact mycelium of *F. oxysporum* in absence of antagonist; (**F**) aggregated hyphae of *F. oxysporum* in presence of antagonist.

The six isolates (Figure 4) which showed the strongest antagonism against the three pathogens were further subjected to secondary screening by paired culture antibiosis to further confirm their antagonistic ability against *C. scovillei*, *C. truncatum* and *F. oxysporum*. The results of this assay indicated that all six isolates were capable of inhibiting the growth of *C. scovillei*, *C. truncatum* and *F. oxysporum*. The isolate AR26 was found to be significantly superior to other isolates in inhibiting mycelial growth of *C. scovillei* (59.63%), *C. truncatum* (61.18%) and *F. oxysporum* (63.58%), respectively followed by the isolate AR10 (Supplementary Figure S1). The isolate AFE2 recorded the lowest percentage of mycelial growth inhibition of the tested pathogens, relative to the other five isolates. Scanning Electron Microscopy (SEM) observations indicated a clear evidence for antifungal activity of isolate AR26 against *C. scovillei*, *C. truncatum* and *F. oxysporum*. The antifungal activity was observed as distinct morphological deformities in pathogen hyphae in the presence of antagonist and hyphae were found to be twisted and shrunk in *C. scovillei*, disintegrated in *C. truncatum*, aggregated into clusters in *F. oxysporum* with reduction in mycelial mat (Figure 5). In contrast, the hyphae in the control plate were dense, intact with regular structure. 

### 3.3. Screening for the Production of Extracellular Lytic Enzymes and Siderophore by the Antagonists

All six actinobacterial isolates were able to produce at least 4 out of 5 hydrolytic enzymes to different degrees. All the isolates tested positive for amylase and cellulase. The isolates AR10, AR26 and AL7 produced siderophore and AR1, AR10, AR26 and AL7 recorded chitinase activity. The isolates AL5 and AFE2 produced protease while all other isolates tested negative for protease activity. The results revealed that the isolates AR10, AR26 and AL7 were positive for siderophore, amylase, cellulase, and chitinase. The isolate AR26 was the most potent antagonist to produce prominently siderophore, cellulase and chitinase (Supplementary Figures S2–S7).

### 3.4. Antifungal Activity of Volatile, Non-Volatile and Thermostable Compounds

**Figure 6:**
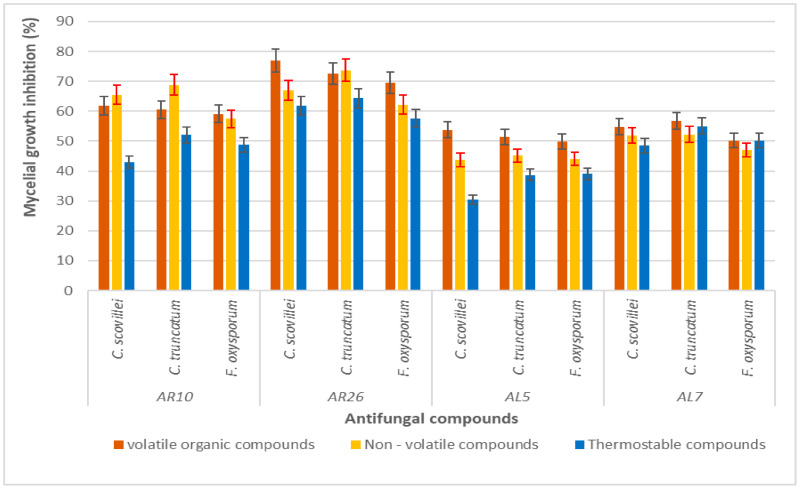
Antifungal activity of volatile, non-volatile and thermostable compounds. Error bars represent the standard deviation of the data set.

All of the four isolates AR10, AR26, AL5 and AL7 apparently produced volatile, non-volatile and thermostable compounds, and significant differences in the antifungal activity against the tested pathogens were observed among the isolates (Figure 6). The volatile compounds of isolates AR10, AR26, and AL5 were found to be more effective than non-volatile and thermostable compounds. The volatile organic compounds of isolate AR26 exhibited the maximum inhibitory effect against *C. scovillei* (77.04%), *C. truncatum* (72.63%) and *F. oxysporum* (69.53%). The thermostable compound of AR26 exhibited the strongest inhibitory action against *F. oxysporum,* followed by AR10.

### 3.5. Assessment of In Vitro Antifungal Traits

The results of the assessment for in vitro antifungal traits revealed that out of the six isolates screened, rhizospheric isolate AR26 showed the highest assessment value of 17 points followed by the isolate AR10 with 15 points. Hence, the actinobacterial isolate AR26 was selected as the most efficient antagonist for further studies (Table 1).

### 3.6. Molecular Confirmation of Actinobacterial Isolates

**Figure 7:**
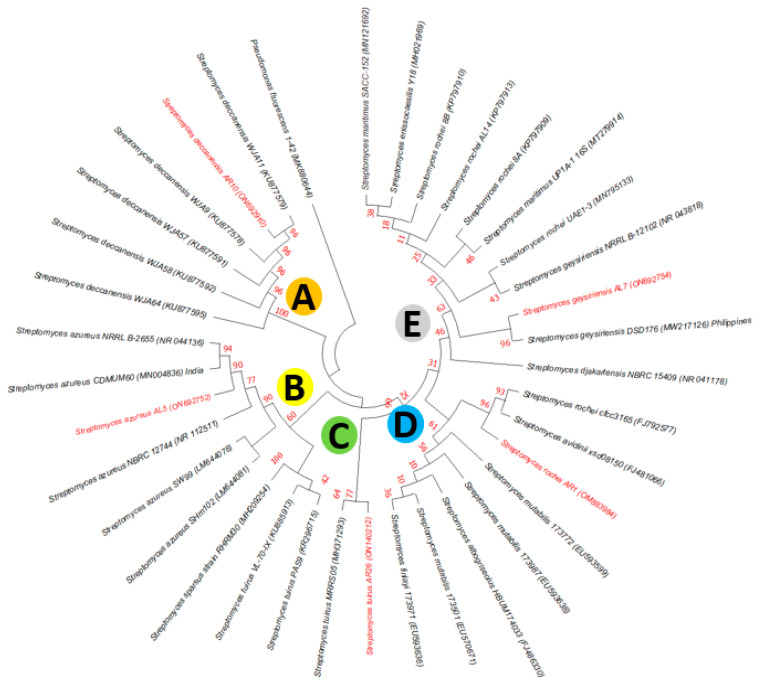
Phylogenetic tree representing the evolutionary relationships of five potent antagonistic actinobacterial isolates isolated from chilli plants. Neighbour joining (NJ) phylogenetic tree constructed from 16S rRNA sequences shows the position of five potent actinobacterial isolates (highlighted in red) and all isolates belong to the genera *Streptomyces*. Bootstrap values (expressed as percentages of 1000 replications) are shown at the nodes. *Pseudomonas fluorescens* 1-42 (MK88064) was used as an outgroup. GenBank accession numbers are given in parenthesis.

The results of the 16S rRNA sequence analysis of the actinobacterial isolates revealed that five isolates were closely affiliated to the genus *Streptomyces.* Isolates AR1, AR10, AL5, AR26 and AL7 exhibited the highest similarity with *Streptomyces rochei*, *Streptomyces deccanensis*, *Streptomyces azureus, Streptomyces tuirus*, and *S. geysiriensis*, respectively (Table 2). Phylogenetic analysis revealed that the isolates under current study formed five different clades (highlighted in red) and were supported with good bootstrap values (Figure 7). Isolate AR10 formed a distinct clade A with *S. deccanensis*, AL5 formed clade B with *S. azureus*, AR26 formed clade C with *S. tuirus,* AR1 formed clade D with *S. rochei,* and AL7 formed clade E with *S. geysiriensis,* with *Pseudomonas fluorescens* as the out group. 

### 3.7. Biocontrol Potential of Liquid Formulation and Methanol Extract of S. tuirus AR26

**Figure 8:**
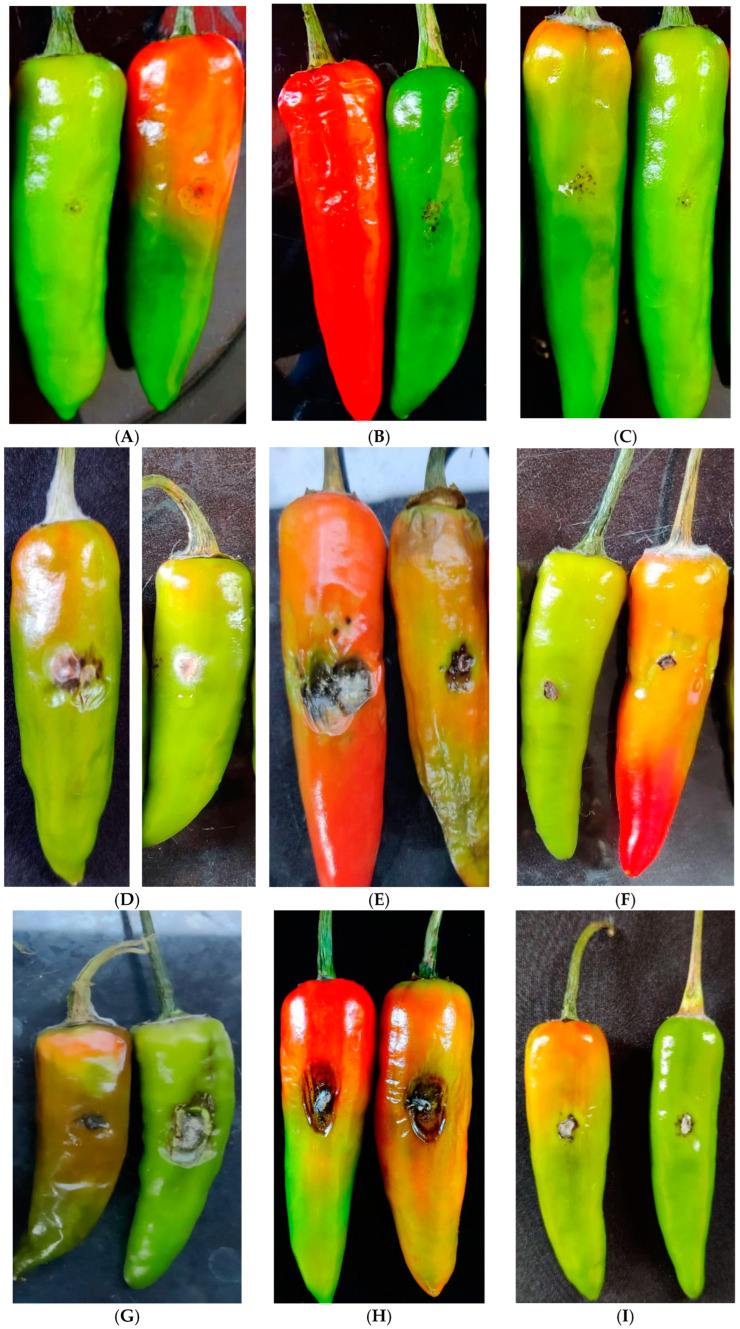

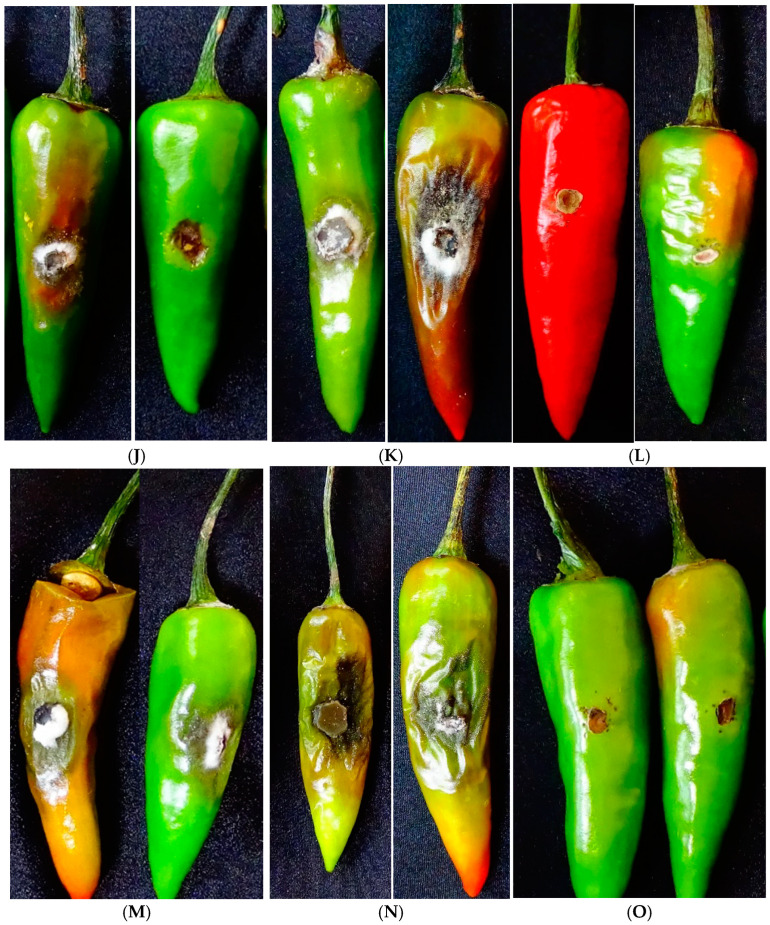
Antifungal efficacy of liquid formulation and methanol extract of *S. tuirus* AR26 against chilli fruit rot pathogens. (**A**) Fruits inoculated with sterile distilled water; (**B**) fruits inoculated with methanol alone; (**C**) fruits inoculated with liquid formulation of *S. tuirus* AR26 at 10 mL/L; (**D**) fruits inoculated with *C. scovillei* and methanol extract of *S. tuirus* AR26; (**E**) fruits inoculated with *C. scovillei* alone; (**F**) fruits inoculated with *C. scovillei* and liquid formulation of *S. tuirus* AR26 at 10 mL/L; (**G**) fruits inoculated with *C. truncatum* and methanol extract of *S. tuirus* AR26; (**H**) fruits inoculated with *C. truncatum* alone; (**I**) fruits inoculated with *C. truncatum* and liquid formulation of *S. tuirus* AR26 at 10 mL/L; (**J**) fruits inoculated with *F. oxysporum* and methanol extract of *S. tuirus* AR26; (**K**) fruits inoculated with *F.oxysporum* alone; (**L**) fruits inoculated with *F. oxysporum* and liquid formulation of *S. tuirus* AR26 at 10 mL/L AR26; (**M**) fruits inoculated with *C. scovillei*, *C. truncatum*, *F. oxysporum* and methanol extract of *S. tuirus* AR26; (**N**) fruits inoculated with *C. scovillei*, *C. truncatum*, *F. oxysporum* alone; (**O**) fruits inoculated with *C. scovillei*, *C. truncatum*, *F. oxysporum* and liquid formulation of *S. tuirus* AR26 at 10 mL/L.

Healthy chilli fruits inoculated with *C. scovillei*, *C. truncatum*, *F. oxysporum* and the co-inoculation of three pathogens produced typical fruit rot symptoms in the form of lesions of up to 2.5, 2.2, 2.6 and 2.9 cm, respectively, seven days after inoculation with the pathogens. Fruits that were not inoculated with the pathogens (healthy control) did not develop fruit rot symptoms, indicating that *C. scovillei*, *C. truncatum* and *F. oxysporum* were the causative agent of the anthracnose disease. Chilli fruits inoculated with the liquid formulation of *S. tuirus* AR26 caused no symptoms or damage to the fruits, indicating its non-pathogenic nature and biocontrol ability (Table 3). The liquid formulation of *S. tuirus* AR26 at both the concentrations 5 mL/L and 10 mL/L caused significant reductions in disease symptom when compared to the pathogen-inoculated control. Application of the liquid formulation of *S. tuirus* AR26 at 10 mL/L completely (100%) suppressed the fruit rot lesions caused by *C. truncatum*, *F. oxysporum* and *Cscovillei + C. truncatum + F. oxysporum. C. scovillei* inoculated fruits recorded 87.9% disease reduction with a corresponding lesion size of 0.30 cm when compared to the *C. scovillei* inoculated control (2.48 cm) (Figure 8). The liquid formulation at 5 mL/L concentration reduced the lesion size by 70.85%, 82.68%, 67.32% and 77.08%, respectively for *C. scovillei*, *C. truncatum*, *F. oxysporum* and the co-inoculation of all the three pathogens with corresponding lesion size of 0.73 cm, 0.38 cm, 0.85 cm and 0.63 cm. Irrespective of pathogens, the metabolites in the methanol extract of antagonist also had significant inhibitory effect on the suppression of fruit rot lesions on chilli fruits compared to the pathogen-inoculated control (Table 4). However, the percentage inhibition of the fruit rot lesion by the antagonist metabolites was significantly lower than the active culture formulation of *S. tuirus* AR26. Antagonist metabolites reduced the lesions up to 70.10%, 62.45%, 53.08% and 44.85% caused by *C. truncatum*, *C. scoviellei*, *F. oxysporum* and co-infection of three pathogens, respectively.

## 4. Discussion

The use of synthetic fungicides is a common practice among farmers for many decades for the management of chilli fruit rot disease; however, this can cause several ill effects to the environment and living creatures. The most urgent and necessary activity of human society is to eliminate the use of fungicides in food crops [66] like chilli which are directly consumed by people. Hence, protecting crops with safe biocontrol agents will not only address concerns about fungicide residues in fresh and processed products, but also increase the export value of fungicide-free food products in domestic as well as world markets. Furthermore, antagonistic microbe–plant interactions reduce the dependence on chemical pesticides by upto 20% [67]. Native actinobacteria adopting a dual role as a biocontrol agent and biofertilizer is a more sustainable, promising and versatile candidate towards the eco-friendly management of plant disease with multiple benefits to society and the ecosystem as a whole.

Hence, in the present study, around 52 actinobacterial isolates were screened for their antagonistic activity against the chilli fruit rot pathogens. Among which, six isolates AR1, AR10, AR26, AL5, AL7 and AFE2 exerted strong antifungal activity against all three pathogens with an inhibition zone of >2 cm and belonged to the genus *Streptomyces*. It was evidenced from previous literature that several species of *Streptomyces* have emerged as biocontrol agents that are safe alternatives to synthetic fungicides for the management of phytopathogens [68,69]. There is ample scientific evidence indicating the successful interaction of various *Streptomyces* spp. with chilli plants to curtail the infection of fruit rot pathogens both at pre- and post-harvest levels. Shahbazi et al. [70] reported that *Streptomyces rochei* strain P42 displayed the highest inhibitory activity against *C. acutatum*, *C. capsici* and *C. gloeosporioides. S. griseocarneus* R132 inhibited the development of anthracnose symptom in chilli fruits [47], and likewise the application of *S. violaceoruber* fermentation broth reduced the incidence of the chilli anthracnose under greenhouse conditions [30].

The results of the present study also revealed that a higher proportion of native rhizospheric actinobacteria exert strong antagonistic activity against *C. scovillei* and *C. truncatum* compared to phyllospheric and endophytic isolates. Similar results were also highlighted by Shahbazi et al. [70], who reported that out of 66 native rhizosphere strains of streptomycetes, 16 strains showed very strong to moderate inhibition against *C. acutatum*, *C. capsici* and *C. gloeosporioides.* Many researchers have reported that diverse species of actinobacteria are recognized to play a crucial function in the rhizosphere by suppressing pathogenic species, as well as promoting the growth and multiplication of beneficial microbes. *Streptomyces* is one of the most dominant and promising biocontrol bacterial genera of plant diseases which efficiently colonise the plant rhizosphere and are known to produce over two-third of antibiotics with the ability to inhibit a wide range of phytopathogens [71,72]. Hyder et al. [73] stated that eight native rhizospheric bacterial isolates obtained from chilli plants were found to exert antifungal activity against damping pathogen *Phytophthora capsici* in vitro and in vivo.

Based on the dual culture and paired antibiosis assay, the actinobacterial isolate AR26 obtained from chilli rhizosphere, which was subsequently identified as *Streptomyces tuirus*, was found to be the most effective isolate in inhibiting the mycelial growth of all the three tested pathogens. This finding is in accordance with the results of Chaudhry [74] who reported that *S. tuirus* strongly inhibited carrot cavity spot pathogen *Pythium violae* including various other pathogens such as *Phytophthora spinosum*, *Phytopythium helicoides*, *Fusarium oxysporum*, *Fusarium falciforme*, *Fusarium solani*, *Sclerotium rolfsii*, and *Sclerotinia sclerotiorum*. Scanning electron micrographs of the interaction of *S. tuirus* with fruit rot pathogens in the dual culture plate revealed mycelial deformities like shrinkage, distortion and aggregation of *C. scovillei*, *C. truncatum* and * F. oxysporum* hyphae, in contrast to dense, smooth and regular mycelium in the control plate. Xu et al. [75] also observed severe morphological and internal abnormalities such as the shrinkage and aggregation of *Magnaporthe oryzae* hyphae when treated with the culture filtrate of rice endophyte *Streptomyces hygroscopicus* OsiSh-2. 

*S. tuirus* also exhibited positive results for most of the antifungal bioassays under study. It is the most potent antagonist to produce almost all the tested extracellular hydrolytic enzymes, most prominently cellulase and chitinase which are reported to be the important hydrolytic enzymes responsible for the biocontrol ability of an antagonist. High chitinase-producing strains are more antagonistic to fruit-rotting pathogens compared to low-chitinase-producing strains [76]. Jha and Modi [77] and Bhattacharyya et al. [78] pointed out that the genus *Streptomyces* is an efficient producer of various lytic enzymes, which plays an important role in the biological control of plant diseases by degrading the cell wall of phytopathogenic fungi made up of chitins and glucans. It is also evident from the earlier reports that *Streptomyces* spp. are significantly responsible for the suppression of plant diseases through the production of chitinase, glucanase [40] and protease [79]. Shahbazi et al. [70] stated that the production of hydrolytic enzymes, especially chitinases, can be considered as a potential antagonistic mechanism against chilli anthracnose pathogens. Therefore, the production of these enzymes will help to select potential actinobacterial isolates for the biological control of the tested pathogens.

*S. tuirus* AR26 is also a highly efficient synthesizer of siderophore which is considered to be one of the most important mechanisms for the biocontrol of plant pathogens [80], in which the antagonist inhibits pathogen growth by depriving it of the available iron in the environment [81]. Hence, it is possible that the siderophore-producing ability of *S. tuirus* AR26 might also have contributed to the suppression of mycelial growth of all the tested pathogens. It is similar to the finding of Liotti et al. [47] who reported the possible role of siderophore of *S. griseocarneus* R132 in the biocontrol of *F. oxysporum* in chilli.

Volatile, non-volatile and thermostable compounds of the *S. tuirus* isolate AR26 also reported significant antifungal activity, particularly volatile organic compounds, which recorded the maximum antifungal activity against the fruit rot pathogens. Many *Streptomyces* spp. were reported to produce various volatile compounds that were effective against the anthracnose disease in various crops [82]. The volatile compounds from *Streptomyces philanthi* RM-1-138 and *Streptomyces* spp. are highly potent for the biocontrol of chili anthracnose caused by *C. gloeosporioides* PSU-NY8 [14] and cucumber anthracnose caused by *C. orbiculare* [83] respectively in the post- harvest pathosystem. Metabolites produced by *Streptomyces* include bioactive compounds such as macrolide, benzoquinones, aminoglycosides, polyenes, and nucleoside antibiotics that are involved in the suppression of various phytopathogens [84,85].

The results of detached fruit assay revealed that application of active antagonists in the form of a liquid bio-formulation was found to be most effective against all the three pathogens compared to methanol extracts. The active culture of the antagonist *S. tuirus* AR26 in the liquid bio formulation caused a significant reduction in the expression of fruit rot symptom, ranging from 87.9% to as high as 100%. It completely suppressed the expression of symptoms caused by *C. truncatum*, *F. oxysporum* and *C. scovillei* + *C. truncatum*, *F. oxysporum* in chilli fruits, which is approximately 30%, 50% and 55% higher than the suppression by the methanol extract. Our finding is in line with the research findings of Sadeghian et al. [50] who also reported that active antagonists as practical formulations seem more effective compared to crude extracts against the bitter rot of apple fruits caused by *C. gloeosporioides*.

Therefore, the inhibition of fruit rot pathogens observed in this study might be due to the antagonistic potential of *S. tuirus* AR26 through the production of antifungal compounds, siderophores, chitinase or through the synergistic action of all these mechanisms. It has been documented in earlier findings that the antifungal ability of actinobacteria might be due to the synergistic activity of two or more antagonistic mechanisms. Furthermore, Evangelista-Martínez [86] also reported that the *Streptomyces* sp. CACIA-1.46HGO strain inhibited the hyphal growth of many fungal plant pathogens by the production of secondary metabolites, extracellular enzymes and probably by the combined effect of these mechanisms. It is well understood from the findings of Yasmin et al. [87] who reported that the antagonistic activity of *Pseudomonas* spp. E227, E233, Rh323, *Serratia* sp. Rh269 and *Bacillus* sp. might be due to the production of siderophores, lytic enzymes and HCN or the synergistic interaction of these two or with other metabolites.

## 5. Conclusions

The management of chilli fruit rot disease still continues to be the focus of intensive research. Though there are several ways of managing this disease, none of the methods were found to be completely successful when applied alone. Hence a preliminary attempt was made to screen the antifungal activity of native actinobacteria against fruit rot pathogens under in vitro conditions. Current results confirmed the potentiality of native actinobacterial isolate *S.tuirus* AR26 to be exploited as a biointensive component under an integrated disease management strategy. The actinobacteria *S. tuirus* AR26 exhibited multifarious biocontrol mechanisms such as the production of volatile, non-volatile and thermostable compounds, competition for iron through the synthesis of siderophores, and production of extracellular lytic enzymes such as chitinase and cellulases. Hence, *S. tuirus* AR26 has a great scope for evaluating its biocontrol potential against chilli fruit rot disease under field conditions as well against a broad spectrum of post-harvest plant pathogens. Larger investigations in the future will demonstrate such possibilities. As farmers become increasingly aware of the concept of sustainable agriculture and organic farming, use of this actinobacteria based bio-formulation will definitely address concerns about ecologically sustainable and socially acceptable long-term solutions to tackle notorious fruit rot pathogens.

## Figures and Tables

**Table 1:** Actinobacterial isolates for their ability to function as antagonist with various antifungal mechanisms.

S.No	Isolate Code	Antagonistic Activity						Antifungal Mechanisms					Total Assessment Points Out of 24
		Mycelial Growth Inhibition (%)						Halo Zone Diameter (cm)					
		Dual Culture Assay			Paired Antibiosis Assay								
		*C.s*	*C.t*	*F.o*	*C.s*	*C.t*	*F.o*	Siderophore	Amylase	Cellulase	Chitinase	Protease	
1.	AR1	1	1	1	1	1	1	0	1	1	1	0	9
2.	AR10	2	2	1	1	2	2	2	1	1	1	0	15
3.	AR26	2	2	2	2	2	2	2	1	1	1	0	17
4.	AL5	1	1	1	2	1	1	0	1	1	0	1	10
5.	AL7	2	1	1	1	1	1	2	1	1	1	0	13
6.	AFE2	1	1	1	1	1	1	0	1	1	0	1	9

Mycelial Growth inhibition percentage (1 = 30–54.9%; 2 = 55–74.9%; 3 = 75–95%); Lytic enzyme production was evaluated with 1 point and siderophore with 2 points each; *C.s*: *C. scoville*; *C.t*: *C. truncatum*; *F.o*: *F. oxysporum.*

**Table 2:** 16S ribosomal RNA partial sequence analysis of actinobacterial isolates and their closest BLASTN matches with NCBI database supplementary.

S. No.	Isolate Name	Isolation Source	NCBI Accession Number	Base Pair Length	Closest 16S rRNA Sequence Match (BLASTN)		Per Cent Identity (%)
					Organism and Strain	Base Pair Length	
1.	AR1	Rhizosphere, Pudukottai	OM883984	1358 bp	*Streptomyces rochei* AL14	1448	99.70
2.	AR10	Rhizosphere, Karaikal	ON692910	1458 bp	*Streptomyces deccanensis* WJA64	1462	99.73
3.	AR26	Rhizosphere, Salem	ON140212	1432 bp	*Streptomyces tuirus* PAS9	1461	99.72
4.	AL5	Phyllosphere: Leaf, Coimbatore	ON692752	1486 bp	*Streptomyces azureus* NRRL B-2655	1516	100.00
5.	AL7	Phyllosphere: Leaf, Trichy	ON692754	1466 bp	*Streptomyces geysiriensis* DSD176	1466	100.00

**Table 3:** Antifungal efficacy of liquid formulation of *Streptomyces tuirus* AR26 against chilli fruit rot pathogens.

S. No	Treatments/Pathogens	Lesion Diameter (cm)	Per cent Disease Reduction (%)
1.	T1: Healthy (uninoculated) control	0.00	100.00 ^a^ (89.71)
2.	T2: Antagonist inoculated control (10 mL/L)	0.00	100.00 ^a^ (89.71)
3.	T3: *Colletotrichum scovillei* inoculated control	2.48	0.00 ^c^ (0.29)
4.	T4: *Colletotrichum truncatum* inoculated control	2.18	0.00 ^c^ (0.29)
5.	T5: *Fusarium oxysporum* inoculated control	2.60	0.00 ^c^ (0.29)
6.	T6: Co-inoculation of *C. scovillei*, *C. capsici* and *F. oxysporum*	2.88	0.00 ^c^ (0.29)
7.	T7: *C. scovillei* + *S. tuirus* (5 mL/L)	0.73	70.85 ^d^ (57.34)
8.	T8: *C. scovillei* + *S. tuirus* (10 mL/L)	0.30	90.32 ^b^ (80.17)
9.	T9: *C. truncatum* + *S. tuirus* (5 mL/L)	0.38	82.68 ^c^ (65.75)
10.	T10: *C. truncatum* + *S. tuirus* (10 mL/L)	0.00	100.00 ^a^ (89.71)
11.	T11: *F. oxysporum* + *S. tuirus* (5 mL/L)	0.85	67.32 ^d^ (55.41)
12.	T12: *F. oxysporum* + *S. tuirus* (10 mL/L)	0.00	100.00 ^a^ (89.71)
13.	T13: Co-inoculation of *C. scovillei*, *C. capsici* and *F. oxysporum* + *S. tuirus* (5 mL/L)	0.63	77.08 ^c,d^ (61.53)
14.	T14: Co-inoculation of *C. scovillei*, *C. capsici* and *F. oxysporum* + *S. tuirus* (10 mL/L)	0.00	100.00 ^a^ (89.71)
	CD (0.05)	0.167	8.040
	SE (d)	0.082	3.960

The values are the mean of three replications. The means in a column followed by the same superscript letters are not significantly different at *p* = 0.05. Values in parenthesis are arc sine transformed.

**Table 4:** In vivo antifungal efficacy of methanol extract of *S. tuirus* AR26 against chilli fruit rot pathogens.

S. No	Treatments/Pathogens	Lesion Diameter (cm)	Per cent Disease Reduction (%)
1.	T1: Healthy (uninoculated) control	0.0	100.00 ^a^ (89.71)
2.	T2: Methanol extract inoculated control	0.0	100.00 ^a^ (89.71)
3.	T3: *C. scovillei* inoculated control	2.93	0.00 ^f^ (0.29)
4.	T4: *C. truncatum* inoculated control	2.50	0.00 ^f^ (0.29)
5.	T5: *F. oxysporum* inoculated control	2.35	0.00 ^f^ (0.29)
6.	T6: Co-inoculation of *C. scovillei*, *C. capsici* and *F. oxysporum*	3.40	0.00 ^f^ (0.29)
7.	T7: *C. scovillei* + Methanol extract of *S. tuirus*	1.10	62.45 ^C^ (52.22)
8.	T8: *C. truncatum* + Methanol extract of *S. tuirus*	0.75	70.10 ^b^ (56.99)
9.	T9: *F. oxysporum* + Methanol extract of *S. tuirus*	1.10	53.08 ^d^ (46.79)
10.	T10: Co-inoculation of *C. scovillei*, *C. capsici* and *F. oxysporum* + methanol extract of *S. tuirus*	1.88	44.85 ^e^ (46.04)
	CD (0.05)	0.326	3.129
	SE (d)	0.159	1.525

The values are the mean of three replications. The means in a column followed by same superscript letters are not significantly different at *p* = 0.05. Values in parenthesis are arc sine transformed.

## Data Availability

The dataset supporting the conclusions of this article are included within the article and its additional files.

## Supplementary Materials

The following are available online at https://www.mdpi.com/article/10.3390/life13020426/s1, Figure S1: Paired antibiosis assay for antifungal activity of actinobacterial isolates against chilli fruit rot pathogens; Figure S2: Relative ability of actinobacterial isolates to produce siderophore and extracellular hydrolytic enzymes; Figure S3: Screening of actinobacterial isolates for amylase activity; Figure S4: Screening of actinobacterial isolates for cellulase activity; Figure S5: Screening of actinobacterial isolates for chitinase activity; Figure S6: Screening of actinobacterial isolates for protease activity; Figure S7: Screening of actinobacterial isolates for siderophore production; Table S1: In vitro antifungal activity of actinobacterial isolates against chilli fruit rot pathogens.
