# Supplementary material for: Exploring the Potentiality of Native Actinobacteria to Combat the Chilli Fruit Rot Pathogens under Post-Harvest Pathosystem
Source: Life (Basel). 2023 Feb 2;13(2):426. doi: 10.3390/life13020426 (PMC9959883; doi:10.3390/life13020426)

Supplementary Table S1: *In vitro* antifungal activity of actinobacterial isolates against chilli fruit rot pathogens

S. No.	Source	Isolate code	*Colletotrichum scovillei*			*Colletotrichum truncatum*			*Fusarium oxysporum*		
			MG (cm)	ZI (cm)	PIMG (%)	MG (cm)	ZI (cm)	PIMG (%)	MG (cm)	ZI (cm)	PIMG (%)
1.	Rhizosphere	AR1	4.07	2.4	54.46 (47.56)	4.43	2.3	50.58 (45.32)	4.57	2.1	49.26 (44.58)
2.		AR2	4.90	1.3	45.13 (42.22)	5.33	1.6	40.54 (39.53)	4.53	1.4	49.63 (44.79)
3.		AR3	4.80	1.1	46.25 (42.85)	5.47	0.5	39.06 (38.65)	6.27	0.4	30.37 (33.43)
4.		AR4	4.30	2.1	51.85 (46.07)	4.47	2.1	50.20 (45.11)	4.67	1.3	48.15 (43.94)
5.		AR5	5.17	1.3	42.14 (40.48)	5.20	1.3	42.03 (40.40)	7.43	0.9	17.41 (24.65)
6.		AR6	4.83	1.1	45.88 (42.63)	6.23	0.4	30.51 (33.48)	6.73	1.5	25.19 (30.11)
7.		AR7	6.07	0.5	32.06 (34.47)	6.10	1.2	32.00 (34.43)	5.30	1.7	41.11 (39.87)
8.		AR8	5.60	0.6	37.29 (37.64)	6.60	1.0	26.42 (30.91)	6.37	0.4	29.26 (32.76)
9.		AR9	4.97	1.5	44.38 (41.75)	5.67	1.2	36.83 (37.35)	5.77	0.8	35.93 (36.81)
10.		AR10	3.67	2.7	58.94 (50.17)	3.93	2.4	56.15 (48.52)	4.23	2.6	52.96 (46.70)
11.		AR11	4.53	2.0	49.23 (44.56)	5.13	1.1	42.7 (40.82)	5.43	1.1	39.63 (39.00)
12.		AR12	4.97	1.4	44.38 (41.77)	5.63	1.4	37.20 (37.57)	5.00	1.5	44.44 (40.73)
13.		AR13	4.60	1.2	48.49 (44.13)	7.13	0.0	20.48 (26.88)	4.80	1.5	46.67 (43.09)
14.		AR14	4.80	1.5	46.25 (42.85)	8.03	0.3	10.44 (18.80)	8.27	0.0	8.15 (16.44)
15.		AR15	4.83	1.6	45.88 (42.66)	5.53	1.2	38.31 (38.23)	4.53	0.9	49.63 (44.79)
16.		AR16	4.90	0.9	45.13 (42.21)	6.67	0.3	25.68 (30.42)	5.93	1.2	34.07 (35.69)
17.		AR17	5.57	0.0	37.66 (37.86)	7.73	0.0	13.79 (21.66)	8.03	0.0	10.74 (19.09)
18.		AR18	4.83	1.3	45.88 (42.64)	6.20	0.0	30.88 (33.73)	6.73	0.5	25.19 (30.10)
19.		AR19	7.93	0.0	11.16 (19.54)	8.50	0.0	5.24 (13.12)	6.87	0.0	23.70 (29.12)
20.		AR20	7.87	0.5	11.91 (20.18)	5.53	1.2	38.31 (38.23)	4.60	2.1	48.89 (44.36)
21.		AR21	6.17	0.8	30.94 (33.78)	7.03	0.4	21.59 (27.67)	5.03	1.1	44.07 (41.58)
22.		AR22	8.33	0.4	6.68 (14.93)	7.17	0.0	20.10 (26.61)	6.27	0.8	30.37 (33.43)
23.		AR23	5.43	1.5	39.16 (38.74)	5.17	1.2	42.40 (40.61)	7.33	0.1	18.52 (25.46)
24.		AR24	5.67	1.6	36.54 (37.21)	5.10	1.4	43.14 (41.05)	4.83	2.1	46.30 (42.88)
25.		AR25	4.23	2.7	52.59 (46.49)	4.33	1.4	51.69 (45.96)	4.30	2.2	52.22 (46.28)
26.		AR26	2.87	3.2	67.90 (55.51)	3.30	2.8	63.21 (52.65)	3.57	2.7	60.37 (50.99)
27.	Phyllosphere: Leaf	AL1	4.40	0.8	50.73 (45.42)	5.80	0.6	35.34 (36.46)	5.43	1.7	39.63 (38.98)
28.		AL2	7.73	0.0	13.40 (21.46)	5.27	1.2	41.29 (39.97)	6.10	0.8	32.22 (34.58)

29.		AL3	8.33	0.0	6.68 (14.99)	6.07	0.0	32.37 (34.66)	7.57	0.0	15.93 (23.47)
30.		AL4	5.03	0.9	43.64 (41.35)	5.20	1.1	42.03 (40.40)	5.43	1.4	39.63 (39.00)
31.		AL5	4.13	2.3	53.71 (47.13)	4.73	2.1	47.23 (43.40)	4.40	2.4	51.11 (45.64)
32.		AL6	6.73	0.5	24.60 (29.73)	5.23	1.4	41.66 (40.18)	7.53	0.0	16.30 (23.80)
33.		AL7	3.93	2.3	55.95 (48.43)	4.47	2.2	50.20 (45.11)	4.20	2.3	53.33 (46.91)
34.		ASt1	7.31	0.0	17.51 (24.69)	4.87	2.0	45.75 (42.54)	6.07	0.7	32.59 (34.80)
35.	Phyllosphere: Stem	ASt2	7.03	0.4	21.24 (27.44)	6.20	0.1	30.88 (33.73)	6.73	0.8	25.19 (30.11)
36.		ASt3	4.40	1.7	50.73 (45.42)	5.53	1.2	38.31 (38.23)	5.17	1.0	42.59 (40.73)
37.		ASe1	6.63	1.6	25.72 (30.49)	5.27	1.5	41.29 (39.96)	5.07	1.2	43.70 (41.35)
38.	Phyllosphere: Seed	ASe2	6.43	0.8	27.96 (31.91)	5.57	1.3	37.94 (38.00)	5.27	1.1	41.48 (40.09)
39.		ASe3	7.03	0.0	21.24 (27.42)	5.63	0.8	37.20 (37.55)	5.37	1.0	40.37 (39.44)
40.		AF1	6.20	1.3	30.57 (33.57)	5.60	1.2	37.57 (37.77)	4.10	1.5	54.44 (47.55)
41.	Phyllosphere: Fruits	AF2	6.17	1.1	30.94 (33.79)	5.33	1.2	40.54 (39.51)	5.50	0.8	38.89 (38.58)
42.		AF3	5.33	0.7	40.28 (39.40)	5.10	1.3	43.14 (41.05)	5.33	1.4	40.74 (39.66)
43.	Endophyte: Leaf	ALE1	8.50	0.0	4.82 (11.90)	5.80	0.9	35.34 (36.45)	4.77	1.5	47.04 (43.30)
44.		ALE2	5.47	1.0	38.78 (38.52)	5.73	0.8	36.08 (36.90)	6.00	0.5	33.33 (35.26)
45.		ALE3	5.90	0.6	33.93 (35.63)	6.17	0.5	31.25 (33.97)	4.87	1.5	45.93 (42.66)
46.		ALE4	6.53	0.7	26.84 (31.22)	5.33	0.7	40.54 (39.49)	6.27	0.7	30.37 (33.42)
47.	Endophyte: Stem	AStE1	5.27	1.6	41.02 (39.83)	5.10	1.2	43.14 (41.05)	7.23	0.3	19.63 (26.28)
48.	Endophytes: Seed	ASeE2	7.03	0.2	21.24 (27.45)	5.33	1.3	40.54 (39.54)	5.80	1.3	35.56 (36.60)
49.		ASeE3	5.87	0.8	34.30 (35.85)	5.63	0.0	37.20 (37.54)	7.03	0.0	21.85 (27.86)
50.		AFE1	7.23	0.0	19.00 (25.82)	5.60	0.0	37.57 (37.77)	8.23	0.0	8.52 (16.88)
51.	Endophytes: Fruit	AFE2	4.50	2.1	49.61 (44.77)	4.77	2.1	46.86 (43.19)	4.50	2.1	50.00
52.		AFE3	7.17	0.4	19.75 (26.319)	5.30	1.4	40.91 (39.74)	5.67	1.1	37.04 (45.00)
53.		Control	8.93	0.0	54.46	8.97	0.0	50.58 (0.29)	9.00	0.0	0.00
54.		SE(d)	0.036	0.049	1.481	0.040	0.064	1.366	0.046	0.062	1.589
55.		CD	0.071	0.098	2.941	0.080	0.128	2.713	0.091	0.123	3.156

Values are mean of three replications. Means in a column followed by same superscript letters are not significantly different according to Duncan's multiple range test at *P = 0.05*. Values in parenthesis are arc sine transformed; **MG:** Mycelial growth of the pathogen **IZ:** Inhibition zone in cm

PIMG: Per cent inhibition of mycelial growth

Supplementary Figure S1: Paired antibiosis assay for antifungal activity of actinobacterial isolates against chilli fruit rot pathogens

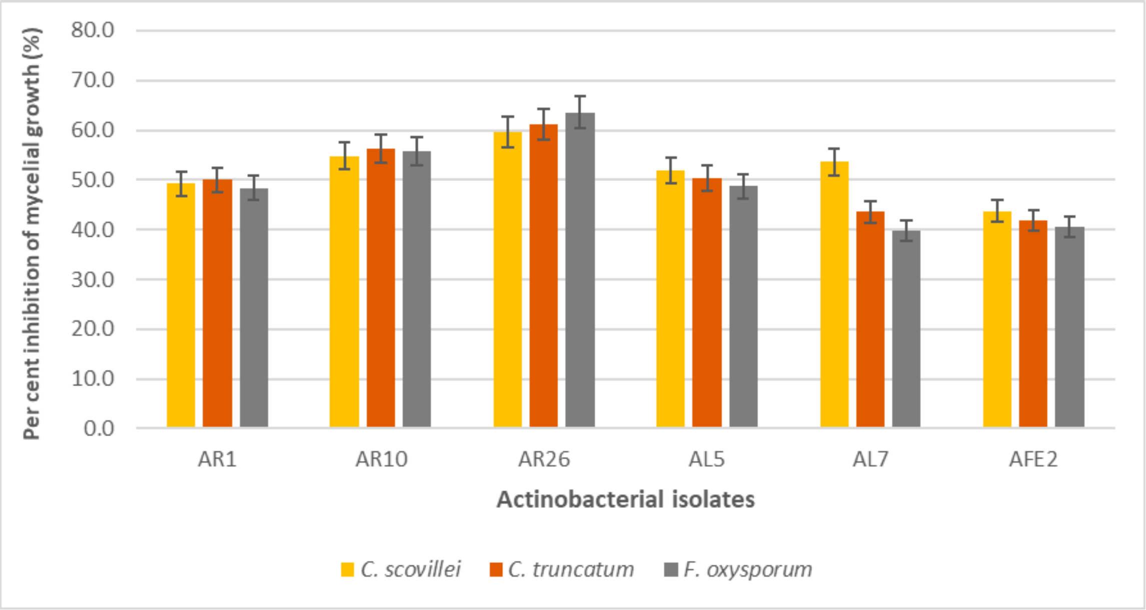

Supplement Figure S2: Relative ability of actinobacterial isolates to produce siderophore and extracellular hydrolytic enzymes

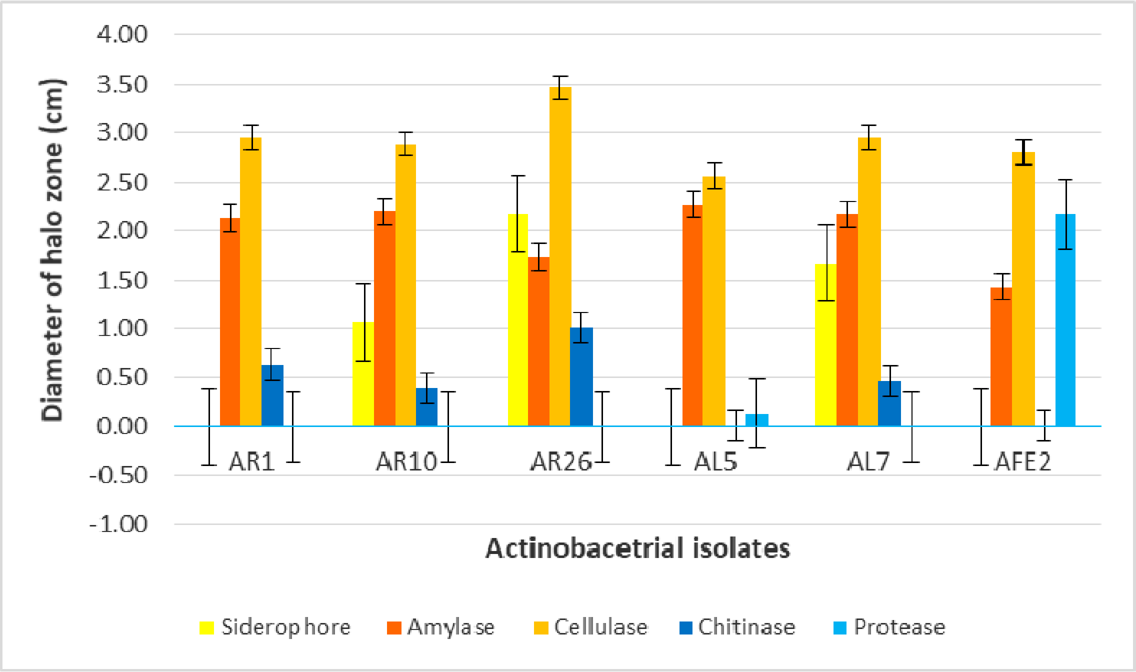

Supplementary Figure S3: Screening of actinobacterial isolates for amylase activity

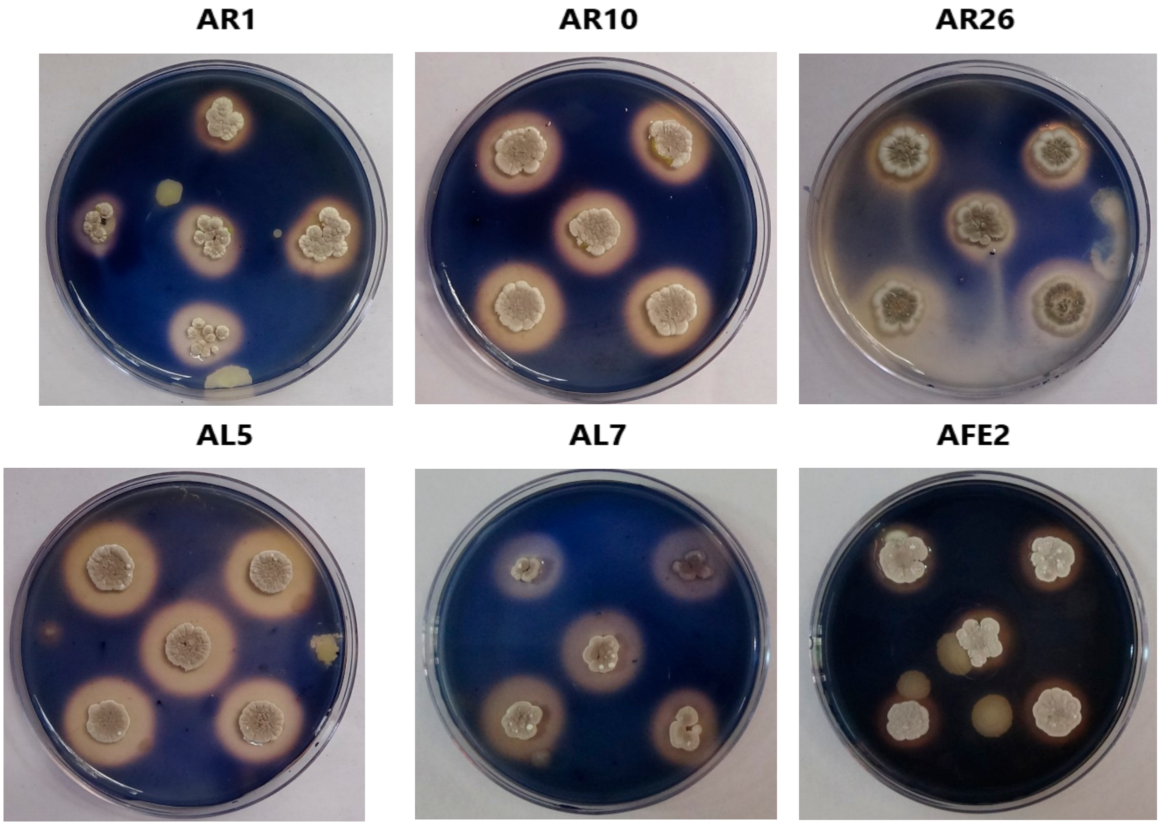

Supplementary Figure S4: Screening of actinobacterial isolates for cellulase activity

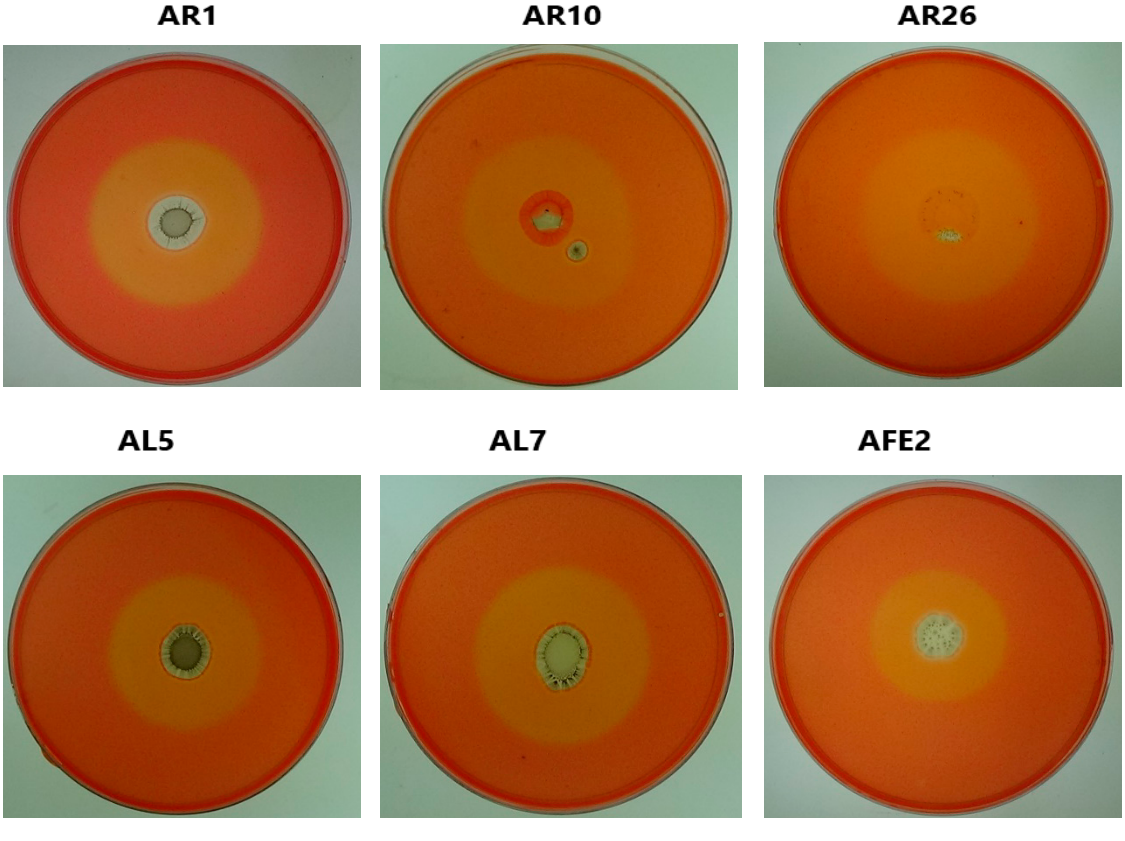

Supplementary Figure S5: Screening of actinobacterial isolates for chitinase activity

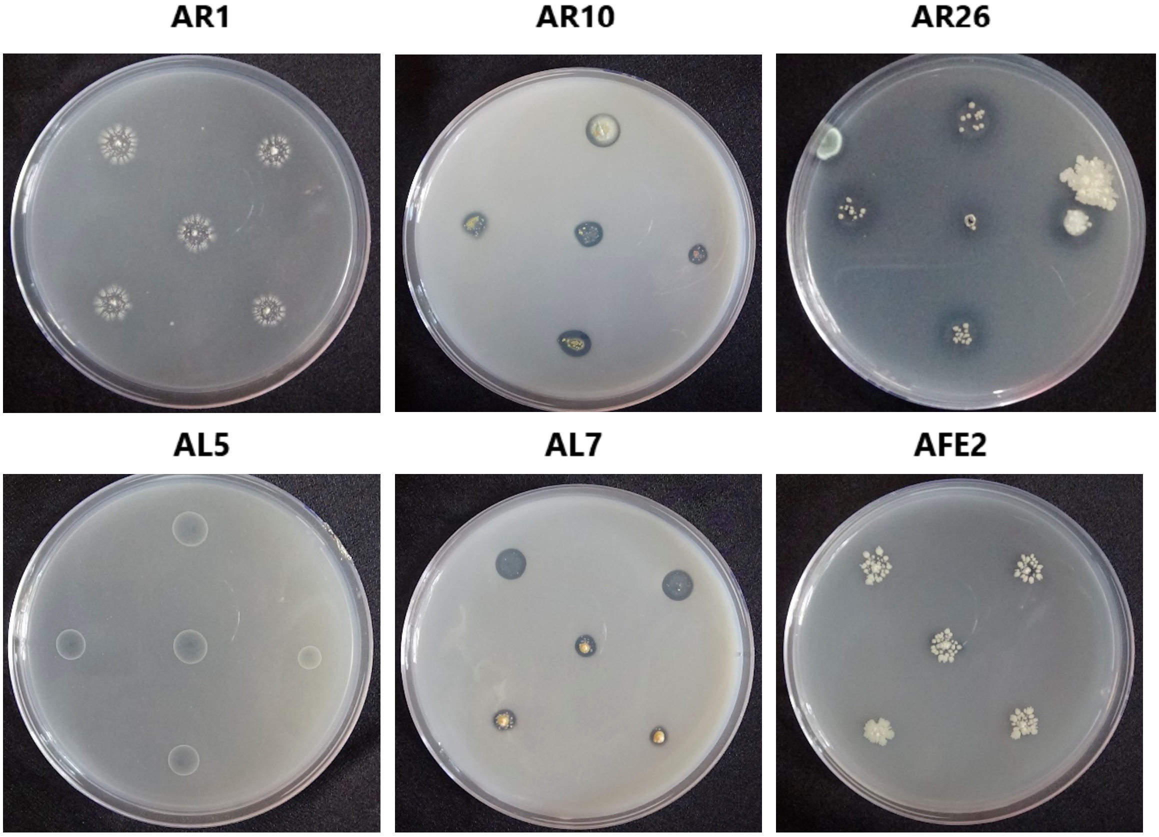

Supplementary Figure S6: Screening of actinobacterial isolates for protease activity

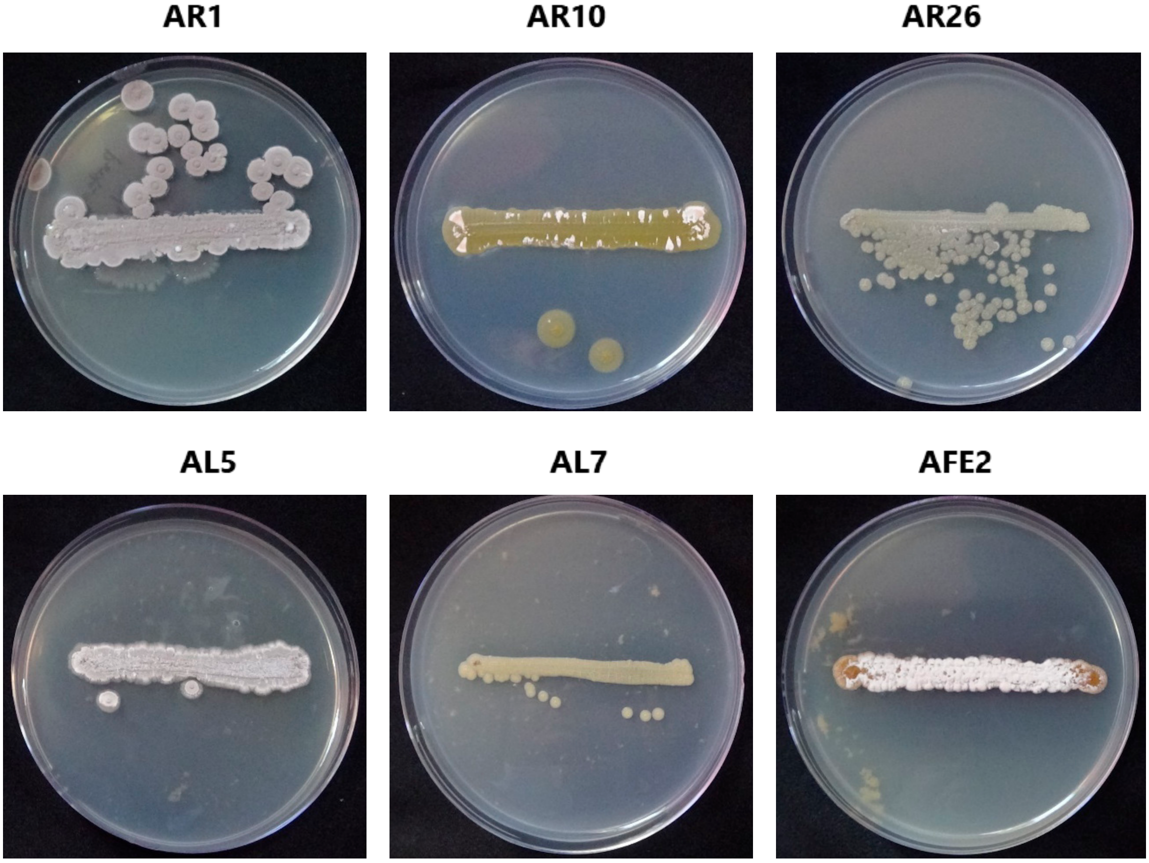

Supplementary Figure S7: Screening of actinobacterial isolates for siderophore production

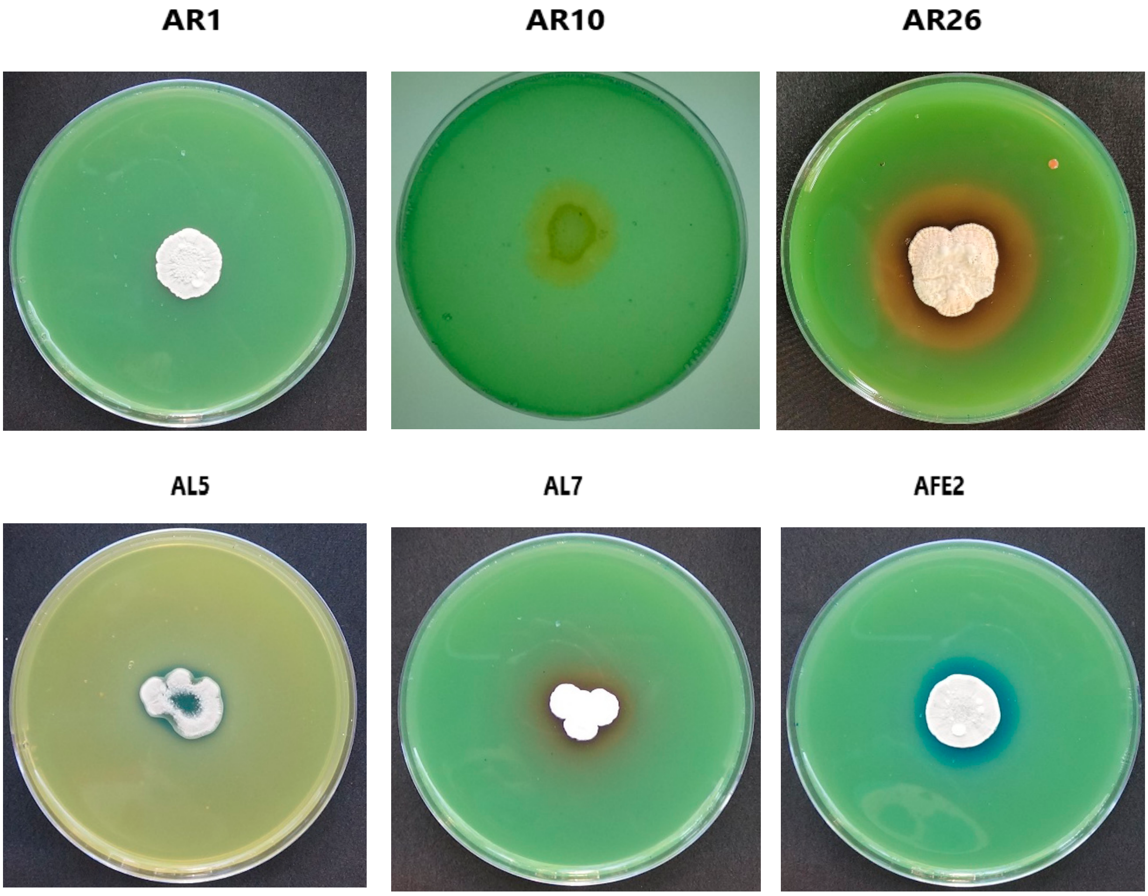

Supplement: Supplementary file 1 [file life-13-00426-s001.zip › life-2093779-supplementary.pdf]
